# Global Perspectives on Mycotoxin Reference Materials (Part I): Insights from Multi-Supplier Comparison Study Including Aflatoxin B1, Deoxynivalenol and Zearalenone

**DOI:** 10.3390/toxins16090397

**Published:** 2024-09-17

**Authors:** David Steiner, Tibor Bartók, Michael Sulyok, András Szekeres, Mónika Varga, Levente Horváth, Helmut Rost

**Affiliations:** 1 LVA GmbH, Magdeburggasse 10, 3400 Klosterneuburg, Austria; helmut.rost@lva.at; 2 Fumizol Ltd., Kisfaludy u. 6/B, H-6725 Szeged, Hungary; tibor.bartok@fumizol.hu (T.B.); levente.horvath@fumizol.hu (L.H.); 3 Department of Agrobiotechnology IFA-Tulln, Institute of Bioanalytics and Agro-Metabolomics, University of Natural Resources and Life Sciences, Vienna, Konrad Lorenz-Strasse 20, 3430 Tulln, Austria; michael.sulyok@boku.ac.at; 4 Department of Biotechnology and Microbiology, University of Szeged, Közép fasor 52, 6726 Szeged, Hungary; szandras@bio.u-szeged.hu (A.S.); vargam@bio.u-szeged.hu (M.V.)

**Keywords:** quality control, certificate of analysis, ISO 17034, impurity, stability

## Abstract

We conducted a comprehensive examination of liquid mycotoxin reference standards. A total of 30 different standards were tested, each containing 10 samples of three distinct substances: Aflatoxin B1, Deoxynivalenol, and Zearalenone. The standards were sourced from 10 different global market leading manufacturers. To facilitate comparison, all the standard sets were adjusted to the same concentration level. The standards were analyzed using the techniques LC-MS/MS, HPLC-DAD, and LC-HRMS to assess their quality attributes. Regarding the validation of the reference values, it was observed that 30% of the suppliers provided reference standards that were either below the lower acceptance limit or above the higher acceptance limit, confirmed by both the LC-MS/MS and HPLC-DAD methods. Furthermore, a total of 12 impurities were found in the DON standards, 10 in the AFB_1_ standards, and 8 in the ZON standards, distributed across all the suppliers. Therefore, this study suggests relevant adjustments to the ISO 17034 standard, proposing that the purity of a raw material should be uniformly based on q-NMR analysis, as most manufacturers state the purity of their certificates is determined using HPLC-UV or LC-MS/MS. Liquid standards with a shelf life of ≤1 year should not exceed an uncertainty of 3%. Standards that have a longer shelf life should not have more than 5% uncertainty. This study also emphasizes the importance of stability. The standards should undergo continuous long-term monitoring; otherwise, products may exhibit a target value of only 80%, as seen in one instance. It is also recommended to include proof of HPLC and LC-MS/MS analyses on the certificate of each released batch of a final product.

## 1. Introduction

Mycotoxins as secondary metabolites are produced by toxinogenic filamentous fungi, and these compounds can occur in different food matrices [1,2,3]. The European Union has set official limit values for food contaminants, including mycotoxins, which, for many years, were laid down in EU Regulation 1881/2006 [4]. This has recently been replaced by EU Regulation 2023/915 [5]. Undoubtedly, mycotoxins are among the most important food contaminants in addition to residues. This EU Regulation currently regulates the maximum levels of 13 mycotoxins, including Aflatoxin B1 (AFB_1_), total Aflatoxin (AFB_1_ + AFB_2_ + AFG_1_ + AFG_2_), Aflatoxin M1, Ochratoxin-A (OTA), Fumonisin B_1_ + B_2_ (FB1 + FB2), Deoxynivalenol (DON), Zearalenone (ZON), T-2, HT-2, and Patulin. These regulations are continuously revised by the EU with the help of the European Food Safety Authority (EFSA) based on the growing knowledge and the results of circular mycotoxin tests. The newly established limits are always incorporated into the original regulation, in this case, in EU Regulation 2023/915.

We estimate that there may be from 5000 to 10,000 private, academic, and official laboratories worldwide that are required to regularly test the possible mycotoxin contamination of various food raw materials and foodstuffs using accredited methods. Accredited status means that these laboratories must use certified reference material (CRM) solutions for the accurate qualitative and quantitative determination of mycotoxins. The manufacturers and distributors of CRM solutions have a great responsibility to ensure that the purity values stated in the certificate accompanying their products are accurate. If the solution of a CRM is less pure than that indicated in the certificate, quantitative evaluation after instrumental measurement may give an incorrect result and, in the case of an official laboratory, the food chain control authority may incorrectly impose a sanction on the company concerned.

High-Performance Liquid Chromatography (HPLC)/Ultra-Performance Liquid Chromatography (UPLC) separation with the use of a general detector, Ultraviolet/Diode Array Detection (UV/DAD), fluorescence detection, and mass spectrometry (FLD and MS) are the most commonly applied methods for the instrumental analysis of mycotoxins [6,7,8,9]. However, none of the HPLC detection methods give absolute data on the purity of the mycotoxin reference materials under testing. The reason is that it is not certain that all the impurities are seen, not to mention that the detector response of the impurities may differ from that of the major component, and if the impurity is unknown, accurate quantification becomes impossible. In the last decade, with the continuous development of nuclear magnetic resonance (NMR) techniques, the so-called quantitative NMR (qNMR) method has entered the world of the quality control of organic compounds. The qNMR technique, in fact, is a proton NMR (1H NMR, qHNMR) assay technique, where the integral value of the component examined is compared to the integral value of the certified qNMR internal standard with known purity. The two compounds (to be tested and ISTD) are very accurately measured on a scale, dissolved in the same known amount of deuterated solvent, and subsequently dispensed into the NMR tube. This detection technique is absolute because the 1H NMR integral value of the component of interest is proportionally reduced by the impurities in the sample to be tested [10,11,12]. To date, only a few qHNMR studies have been published on the determination of mycotoxin purity [13,14,15], but the application of this technique during mycotoxin standard preparation is strongly recommended in the future.

In addition to the technical advancements, the establishment of ISO 17034 was a significant milestone for the certification of reference material producers. [16] Introduced in November 2016, ISO 17034 specifies requirements for the competence of reference material producers, ensuring that the reference materials are produced and certified with high accuracy and consistency. This standard replaced the earlier ISO Guide 34, which had previously governed the certification of reference material producers [17]. ISO Guide 34, introduced in 2000, was a set of guidelines rather than an official standard [18]. It outlined the fundamental requirements for the production and certification of reference materials, but lacked the comprehensive and stringent criteria now found in ISO 17034. The transition from ISO Guide 34 to ISO 17034 provided a more detailed framework, addressing quality assurance, traceability, and documentation in greater depth. ISO 17034 thus represents a more robust and authoritative standard for ensuring the reliability of reference materials across laboratories worldwide. The ISO 17034 standard is complemented by companion documents, such as ISO 33405:2024 “Reference materials—Approaches for characterization and assessment of homogeneity and stability” and ISO 33407:2024 “Guidance for the production of pure organic substance certified reference materials”, alongside others. In our work, the CRM samples were purchased from different manufacturers/distributors at low concentration levels (usually 1–200 µg/mL); thus, it was not possible to perform qHNMR assays on the CRM samples. As a qNMR assay requires at least 1–4 mg of material, it has significance mainly in the preparation of a mycotoxin batch using the standard production process. Therefore, we were interested to see if the CRM certificates included qHNMR measurement data in addition to the quantitative results of the other assays mentioned above.

To our knowledge, no available data on the comparison of mycotoxin reference materials have been published in the scientific literature. Therefore, this publication is a gap-filling one to reveal the quality of the CRM of mycotoxins on the chemical market compared to each other and how they conform with the data as described in their certificates. As this report is not about advertising, rather it is about the scientific comparison of reference materials, all the manufacturers/distributors are listed by code numbers without their names. We intend to publish a series on the CRMs for all mycotoxins with the EU limits. In the first part, we will discuss AFB_1_, DON, and ZON toxins, while AFB_2_, AFG_1_, AFG_2_, AFM_1_, FB1, FB2, OTA, T-2, HT-2, and patulin toxins will be detailed in the following parts.

## 2. Results

### 2.1. Individual Target Values Based on LC-MS/MS and HPLC-DAD

The agreement between the HPLC-DAD and LC-MS/MS measurements was exceptionally strong, with both the methods providing average relative standard deviation (RSD) values well below 3%. The RSD values serve as a measure for the comparability of the methods, underlining the reliability and objectivity of the target value determinations achieved through these techniques.

The 3% criterion, while not universally fixed, is based on general practice and the empirical evidence considered in many scientific publications and analytical method standards. For instance, in the ICH Q2 (R1) *Validation of Analytical Procedures* [19], while it does not specify explicit values for RSD, in practice, RSD values below 3% are often used as an indicator of good precision and repeatability. Similarly, the FDA Guidance for Industry, *Bioanalytical Method Validation* [20], provides general guidelines for precision, where an RSD of less than 15% (and less than 20% at the lower limit of quantification) is considered acceptable. However, for many applications in analytical chemistry, especially in determining target value rates, a much stricter value of 3% is targeted.

Table S3 in the Supporting Information shows a general overview of the RSD and target value data compiled from the HPLC-DAD and LC-MS/MS measurements.

**Figure 1:**
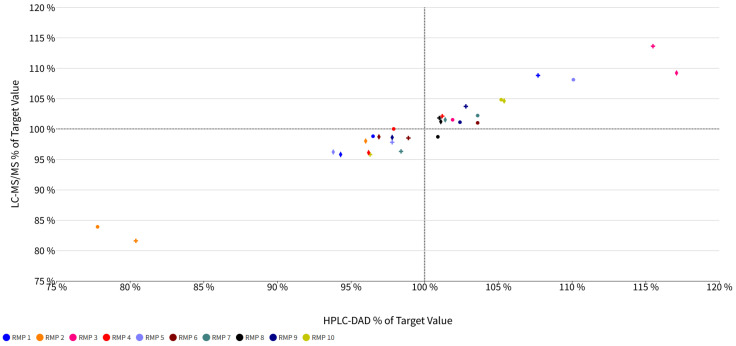
Overview of LC-MS/MS and HPLC-DAD target values in %. Crosses represent Deoxynivalenol, circles indicate Aflatoxin B1, and diamonds represent Zearalenone.

For HPLC-DAD, the average RSD values were 0.77% for AFB_1_, with the individual RSDs ranging from 0.13% to 2.58%; 1.88% for DON, with the individual RSDs ranging from 0.97% to 2.56%; and 0.60% for ZON, with the individual RSDs ranging from 0.44% to 0.81%. Similarly, the LC-MS/MS measurements demonstrated average RSD values of 1.89% for AFB_1_, with the individual RSDs ranging from 0.33% to 5.34%; 0.70% for DON, with the individual RSDs ranging from 0.32% to 1.21%; and 2.04% for ZON, with the individual RSDs ranging from 1.04% to 2.85%. These consistent RSD values, predominantly under the 3% threshold, indicate high repeatability and precision for both the analytical methods. Figure 1 shows a graphical overview of the target values obtained for each standard set.

The target value rates, defined as the average peak area of each standard from three repeat measurements compared to the average peak areas from three repeat measurements of all the replicates in the respective standard set (*n* = 30), serve as a quality measure for each standard. For HPLC-DAD, the target value rates for ABF1 ranged from 77.8% to 110.1%, for DON, they ranged from 80.4% to 115.5%, and for ZON, they ranged from 93.8% to 117.1%. In comparison, the LC-MS/MS measurements showed target value rates for AFB_1_ between 83.9% and 108.1%, for DON from 81.6% to 113.6%, and for ZON between 95.8% and 109.2%.

These findings indicate that both the analytical methods provided comparable and reliable target value rates for the standards tested. The low RSD values across both the methods emphasize the high repeatability and precision of the measurements, ensuring that the determination of target values is both significant and objective.

### 2.2. Verification of the CoA Reference Value

**Figure 2:**
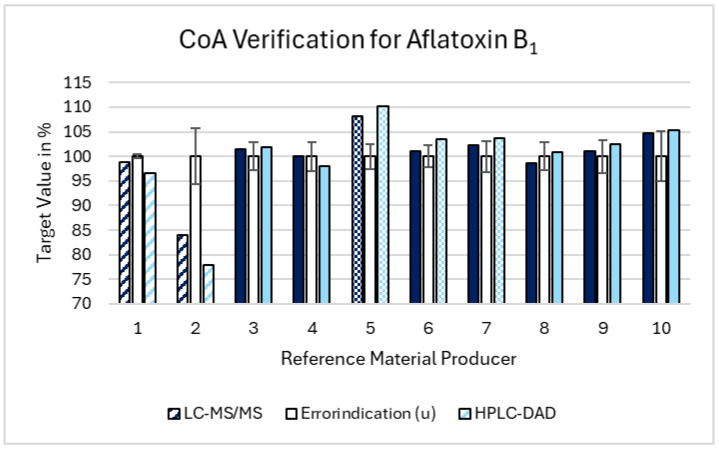
Summary of the Aflatoxin B1 results regarding the validation of the CoA. The acceptance range is depicted by the error indicators (the white bars positioned at the center for each standard/supplier combination) accompanied by their respective error bars. The dashed bars represent the values falling below the CoA acceptance range, while the dotted bars indicate the values exceeding it. The solidly filled bars fall within the acceptance range, meeting the specified criteria.

For AFB_1_, the average combined uncertainty was 3.1%, with a range from 0.4% to 5.7%. Two suppliers provided values below the lower acceptance limit, confirmed by both the LC-MS/MS and HPLC-DAD methods. One supplier exceeded the higher acceptance limit with both the methods, while two additional suppliers surpassed the higher limit based solely on the HPLC-DAD results. Overall, 30% of the suppliers (three out of ten) were confirmed to be outside the acceptance range by both the methods. When considering the deviations detected by either method, 50% of the suppliers (five out of ten) were outside the acceptance range. The results for AFB_1_ are graphically presented in Figure 2.

**Figure 3:**
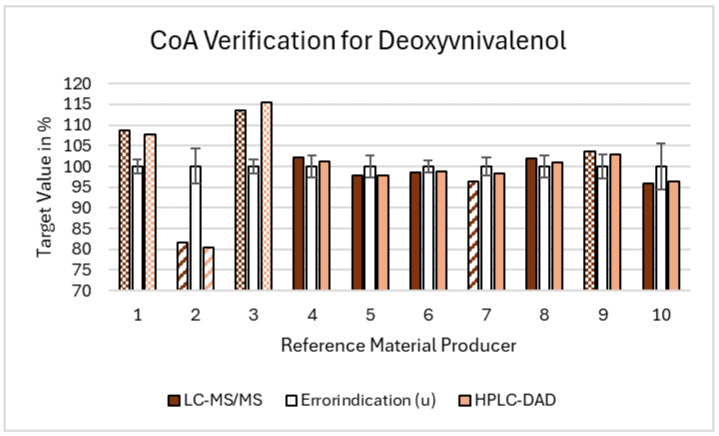
Summary of the Deoxynivalenol results regarding the validation of the CoA. The acceptance range is depicted by the error indicators (the white bars positioned at the center for each standard/supplier combination) accompanied by their respective error bars. The dashed bars represent values falling below the CoA acceptance range, while the dotted bars indicate values exceeding it. The solidly filled bars fall within the acceptance range, meeting the specified criteria.

For DON, the average combined uncertainty was 2.8%, with a range from 1.7% to 5.7%. One supplier provided values below the lower acceptance limit (according to chapter 2.3), confirmed by both the LC-MS/MS and HPLC-DAD methods. Two suppliers exceeded the higher acceptance limit with both the methods, and one additional supplier was outside the range based only on LC-MS/MS results. Thus, 30% of the suppliers (three out of ten) were outside the acceptance range according to both the methods, while 40% (four out of ten) were outside the range when including deviations detected by either method. The results for DON are graphically presented in Figure 3.

**Figure 4:**
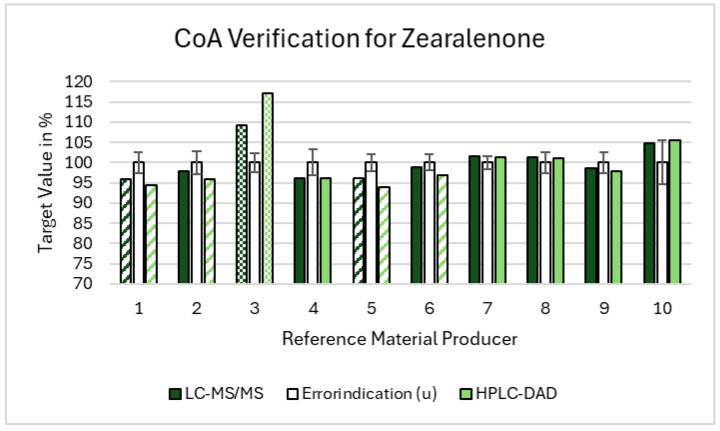
Summary of the Zearalenone results regarding the validation of the CoA. The acceptance range is depicted by the error indicators (the white bars positioned at the center for each standard/supplier combination) accompanied by their respective error bars. The dashed bars represent values falling below the CoA acceptance range, while the dotted bars indicate values exceeding it. The solidly filled bars fall within the acceptance range, meeting the specified criteria.

For ZON, the average combined uncertainty was 2.7%, with a range from 1.6% to 5.4%. Two suppliers provided values below the lower acceptance limit, confirmed by both the LC-MS/MS and HPLC-DAD methods. One supplier exceeded the higher acceptance limit with both the methods, and three additional suppliers were outside the range based on one method (two with HPLC-DAD and one with LC-MS/MS). Thus, 30% of the suppliers (three out of ten) were confirmed to be outside the acceptance range by both the methods, and 60% (six out of ten) were outside the range when considering the deviations detected by either method. The results for ZON are graphically presented in Figure 4.

In summary, across all the three mycotoxins, we observed that 30% of the suppliers provided reference standards that were either below the lower acceptance limit or above the higher acceptance limit, confirmed by both the LC-MS/MS and HPLC-DAD methods. When including the deviations detected by either method, 50% of the suppliers were outside the acceptance range. These discrepancies can be attributed to several factors. The suppliers falling below the lower limit may have stability problems, leading to degradation and lower concentrations of the mycotoxin standards. For example, improper storage conditions or the inherent instability of the compounds can result in such degradation. The suppliers exceeding the higher limit might indicate potential contamination or impurities in the reference materials, which could artificially elevate the measured concentrations. Contamination could occur due to insufficient purification during the production process. The variability in uncertainty specifications highlights the need for standardization and rigorous quality control to ensure reliable reference standards. An overview of all the data, including the uncertainty contributions, is depicted in the Supporting Information within Table S4.

### 2.3. Impurities Determined by LC-HRMS

**Figure 5:**
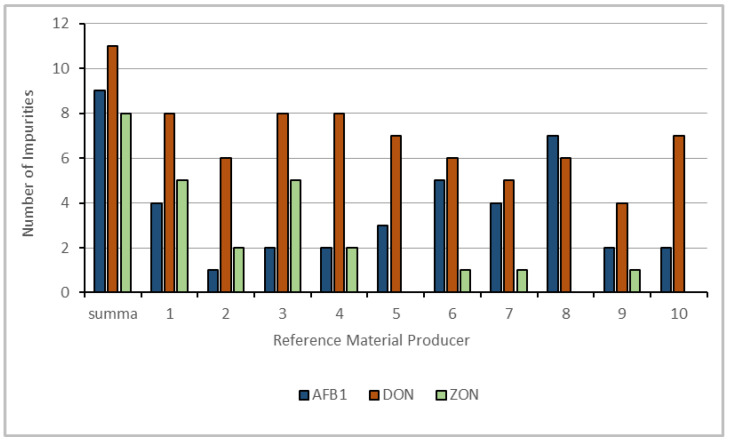
Impurity content of mycotoxin standards.

In total, eleven impurities were found in the DON standards, nine were found in the AFB_1_ standards, and eight were found in the ZON standards, distributed across all the suppliers. A graphical representation of the number of impurities is illustrated in Figure 5.

Detailed results, including information on the molecular mass, the annotation error, the retention time, and fragmentation, can be found in the Supporting Information, Table S5.

In the AFB_1_ standards, Aflatoxin B2 (AFB_2_) and Aflatoxin G1 (AFG_1_) were also found alongside AFB_1_. The detection of these mycotoxins can be achieved, as aflatoxigenic *Aspergillus* species can produce either AFB only or both AFB and AFG mycotoxins. The presence of AFB_2_ and AFG_1_ in the AFB_1_ standards allows for the deduction of a common producer, with the ratio of AFB_2_ to AFG_1_ providing insights into the biosynthetic pathway and potential environmental conditions influencing Aflatoxin production.

In the DON standards, ZON was identified alongside DON. The co-occurrence of these mycotoxins can be attributed to the biosynthetic pathway of DON-producing *Fusarium* species, which have the enzymatic machinery to synthesize ZON and B-type trichothecenes.

Among the impurities detected in the ZON standards, compounds C18H24O5 (RT = 18.35 min) and C18H24O5 (RT = 20.91 min) were identified, which may correspond to Zearalanone and alpha (beta)-Zearalenol, respectively. These compounds are the major metabolites of ZON. The characteristic mass spectral fragments of these compounds are consistent with those reported in the literature. The presence of these metabolites alongside ZON in the standards suggests a shared biosynthetic pathway. Moreover, their ratio could provide valuable information about the fungal strain and environmental conditions, further aiding in the identification of the common producer.

**Figure 6:**
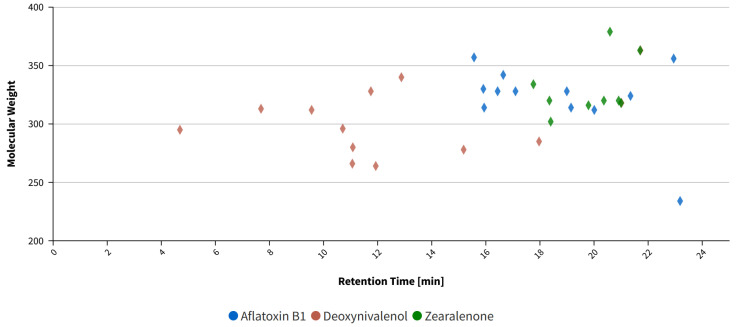
The distribution of impurities detected in each mycotoxin standard (AFB_1_, ZEN, and DON). Each colored dot corresponds to an impurity found within the respective standard, as indicated by the color code. The y-axis represents the molecular weight of the impurities, while the x-axis shows their retention time in minutes (min). The impurities are color-coded to match the standard in which they were identified. A detailed list of the impurities is provided in Supporting Table S5.

Figure 6 illustrates the distribution of all the impurities plotted against the retention time.

During the cultivation of aflatoxigenic *Aspergillus* species, both the AFB and AFG mycotoxins can be produced depending on the strain and the environmental conditions. The ratios of AFB_2_ to AFG_1_ in the AFB_1_ standard likely reflect the natural variability in these production processes. Similarly, the co-occurrence of DON and ZON in the DON standards can be attributed to the biosynthetic capabilities of *Fusarium* species, where both B-type trichothecenes and ZON are synthesized simultaneously.

In terms of purification, separating the closely related mycotoxins can be challenging, especially when they share similar chemical properties. Incomplete purification can result in the carryover of these related compounds into the final standard. The observed impurities in the ZON standards, such as Zearalanone and alpha (beta)-Zearalenol, also suggest that these compounds were not fully separated during purification, likely due to their structural similarity to ZON. Detailed analysis of the production and purification steps would be necessary to minimize such co-contaminations, ensuring higher purity in the final standards.

## 3. Discussion

This study emphasizes the critical need for enhancing the ISO 17034 standard by incorporating more rigorous and uniform procedures for the purity assessment of mycotoxin reference materials. The current practice predominantly reliant on methods such as HPLC-UV or LC-MS/MS, which exhibit significant limitations in obtaining absolute purity data. To address these challenges, it is proposed that the purity assessment of raw materials should be standardized using q-NMR analysis. Implementing q-NMR analysis as a standard procedure will significantly improve the accuracy and reliability of purity measurements, thus ensuring that the reference materials meet the highest quality standards required for precise and reliable analytical applications.

Uniformity in the specification of uncertainty on CoAs is another essential improvement identified by this study. Presently, there is considerable variability in how different suppliers report uncertainty, leading to inconsistencies that can compromise the reliability of analytical results. To mitigate this issue, it is recommended that the uncertainty for liquid standards with a shelf life of up to one year should not exceed 3%. For the standards with longer shelf lives, uncertainty should be capped at 5%. These thresholds are supported by this study’s findings, which indicate an average uncertainty of 2.3% across all the standards. This approach not only harmonizes the uncertainty specifications, but also ensures that the majority of standards, which typically have a shelf life of one year, are produced and utilized within a stringent and reliable framework.

Furthermore, this study highlights the necessity of continuous, long-term stability monitoring for mycotoxin reference standards. Stability issues can lead to significant degradation, rendering the standards unsuitable for analytical purposes. This investigation revealed instances where the standards exhibited target value recovery rates as low as 80% due to stability degradation. To prevent such occurrences, it is crucial that manufacturers implement rigorous and continuous long-term stability testing protocols. The standards that fail to meet the established stability criteria should not be distributed or used, ensuring that only the highest quality reference materials are available for analytical use.

In addition, this study advocates for the inclusion of HPLC or LC-MS/MS analysis results in the CoAs of all the final liquid mycotoxin reference materials. This addition will greatly enhance transparency and traceability, providing the end-users with comprehensive information regarding the purity and quality of the standards. Including a statement that each released batch has undergone a release testing will further reinforce the reliability and credibility of the reference materials. This practice not only facilitates more informed decision making by users, but also aligns with the broader objective of maintaining stringent quality controls in the production and certification of reference materials.

## 4. Conclusions

The comprehensive examination of mycotoxin reference standards conducted in this study has shed light on critical aspects that require attention and adjustment within the ISO 17034 standard. By evaluating the quality attributes of reference materials from various manufacturers and employing advanced analytical techniques, we have identified several areas where improvements can be made to enhance the reliability and accuracy of mycotoxin analysis.

In conclusion, this study has identified the key areas where relevant adjustments to the ISO 17034 standard can be made to enhance the quality and reliability of mycotoxin reference materials. The key findings include that 30% of the tested standards were outside the acceptable range for reference values, with significant impurities identified across all the samples. This study highlights the need for standardized purity assessments using q-NMR instead of relying solely on HPLC-UV or LC-MS/MS, as is currently the common practice.

Additionally, it is recommended that liquid standards with a shelf life of one year or less maintain an uncertainty below 3%, while those with a longer shelf life should remain under 5%. Ensuring continuous, long-term stability monitoring is crucial to prevent substantial deviations from the target values. Finally, it is suggested to include documented HPLC and LC-MS/MS analyses in the certificates of each released batch to enhance quality and reliability.

By standardizing the analytical methods for purity testing, uncertainty specifications, and stability monitoring protocols, as well as by including information about the release testing in CoAs, we can significantly improve the accuracy and reliability of mycotoxin analysis. These proposed adjustments will contribute to the overall improvement of food and feed safety, safeguarding public health and ensuring compliance with the regulatory standards. Further research and collaboration among stakeholders are needed to implement these adjustments effectively and to promote the continuous improvement of mycotoxin analysis practices.

## 5. Materials and Methods

### 5.1. Reference Standards

In this study, comprehensive examination of liquid mycotoxin reference standards was conducted to determine any disparities in product-related quality attributes. A total of 30 different liquid standards were tested for comparison in this quality study, encompassing 10 samples each of three distinct substances: AFB_1_, DON, and ZON. The standards were sourced from the following manufacturers: CPI International (Santa Rosa, CA, USA), LVA (Klosterneuburg, Austria), Romer Labs (Tulln, Austria), Trilogy (Washington, DC, USA), JRC European Commission (Geel, Belgium), Fermentek (Jerusalem, Israel), Fianovis (Vindry-sur-Turdine, France), Merck Supelco (Laramie, WY, USA), Oskar Tropitzsch (Marktredwitz, Germany), and Pribolab (Singapore, Singapore). Information regarding the individual purities of the raw material was limited. However, when such information was available, it was stated as >99%.

Table S1 within the Supporting Information provides an overview of the standards utilized in this study.

### 5.2. Standardization of Concentrations for Comparative Analysis

To facilitate the comparison of the reference values, all the standard sets were adjusted to the same concentration level. A uniform concentration of 200 µg/L was set for AFB_1_, while DON and ZON were each adjusted to 1000 µg/L. Dilution in acetonitrile/water (20:80) was performed to establish consistent solvent ratios and optimize the balance of organic and inorganic solvents for the optimal peak generation of polar and non-polar compounds. An overview of this dilution process is provided in the Supporting Information, Table S2.

The validity of individual reference values was determined through triplicate measurements per standard set (n = 30) employing both liquid chromatography–mass spectrometry (LC-MS/MS) and HPLC-DAD methodologies. A mean of the 30 data points from each set was calculated to serve as a reference point for the individual evaluation of the standards.

For impurity assessment using liquid chromatography–high resolution accurate mass spectrometry (LC-HRMS), the standards were analyzed without dilution to ensure maximal signal intensity.

### 5.3. Calculation for Verifying Reference Values

To verify the reference values on the certificate of analysis (CoA), a comprehensive approach was taken, considering multiple factors expressed as follows:

$$u=\;\sqrt{{\left(u_{\mathrm{CoA}/2}\right)}^{2}+{\left(u_{\mathrm{MS}/\mathrm{MS}}\right)}^{2}+{\left(u_{\mathrm{DAD}}\right)}^{2}+{\left(u_{\mathrm{prep}}\right)}^{2}}$$

The resulting combined uncertainty (*u*) provides the acceptance range, including acceptable upper and lower limits for each reference value per standard. To verify the reference value, the uncertainty value was added to or subtracted from the reference value stated on the CoA to define the individual acceptance range. If the target value fell outside this range, the standard was considered unverified, indicating potential quality issues. The following factors were integrated into this formula:

*u_CoA_*

This component represents the uncertainty specified on the CoA for each individual measurement. The value provided on the CoA reflects the manufacturer’s or supplier’s assessment of the measurement’s uncertainty. This value was divided by a factor of 2 before being combined with the other uncertainty components below, as it represents expanded uncertainty.

*u_MS/MS_*

This component accounts for the variability observed in the LC-MS/MS triplicate measurements from one vial.

*u_DAD_*

Similar to the *u_MS/MS_* component, this factor accounts for the variability observed in the HPLC-DAD triplicate measurements from one vial.

*u_prep_*

Uncertainty “*u_prep_*” reflects the variability associated with the preparation of standards at the target concentration levels. Due to the inherent limitations in pipetting, the actual concentrations may slightly deviate from the intended values. For example, if a target concentration of 200.0 µg/L results in a final concentration of 199.5 µg/L, this deviation corresponds to 0.25%. These deviations are quantified as “*u_prep_*” and are documented in Table S2 of the Supporting Information.

### 5.4. HPLC-DAD Analysis

HPLC-DAD measurements were conducted at LVA GmbH in Klosterneuburgfor AFB_1_, DON, and ZON utilizing an Agilent 1200 HPLC system equipped with a Diode Array Detector. Chromatographic separation was achieved on a Kinetex 2.6 µm, 100 × 3 mm column employing a gradient elution profile.

For AFB_1_, the gradient elution profile commenced with 100% mobile phase A (methanol/water, 10:90, *v*/*v*) and transitioned linearly to 50% A over 3 min after minute 2. From 5 to 9 min, the gradient shifted to 100% mobile phase B (methanol/water, 97:3, *v*/*v*). This condition was maintained for 3 min before returning to 100% A after 0.1 min, followed by an additional 1.9 min for column equilibration. For DON and ZON, the gradient elution profile began with 90% mobile phase A (H3PO4 0.1%) and transitioned linearly to 50% A over 3 min after minute 2. From 5 to 9 min, the gradient shifted to 100% mobile phase B (acetonitrile). This condition was maintained for 3 min before reverting back to 90% A after 0.1 min and held for an additional 1.9 min for column equilibration.

The flow rate was maintained at 0.5 mL/min, and the injection volume was 20 µL. The column temperature was set at 35 °C, and detection was performed at wavelengths of 365 nm for AFB_1_, 220 nm for DON, and 236 nm for ZON using the DAD. Data acquisition and processing were conducted using Agilent OpenLab 3.6 software.

### 5.5. LC-MS/MS Analysis

The LC-MS/MS method employed in this study utilized a QTrap 5500 MS/MS system (Sciex, Foster City, CA, USA) equipped with a TurboV electrospray ionization (ESI) source coupled with a 1290 series UHPLC system (Agilent Technologies, Waldbronn, Germany), and it was carried out by University of Natural Resources and Life Sciences, Vienna (BOKU), at IFA Tulln. Chromatographic separation was conducted at 25 °C on a Gemini C18 column (150 × 4.6 mm i.d., 5 μm particle size) equipped with a C-18 security guard cartridge (4 × 3 mm i.d., both from Phenomenex, Torrance, CA, USA). Elution proceeded in binary gradient mode at a flow rate of 1000 μL/min, with the mobile phases composed of methanol/water/acetic acid (10:89:1, *v*/*v*/*v*; mobile phase A) and (97:2:1, *v*/*v*/*v*; mobile phase B) [21].

Following a 2 min initial period at 100% A, the proportion of B was linearly increased to 50% within 3 min. Subsequently, B was linearly raised to 100% over 9 min and maintained for 4 min, followed by a 2.5 min column re-equilibration at 100% A. The injection volume was 5 μL. ESI-MS/MS was performed in scheduled multiple reaction monitoring mode both at positive and negative polarities over two separate chromatographic runs.

### 5.6. LC-HRMS Analysis

LC-HRMS measurements were conducted at the University Szeged in Hungary by using a Dionex Ultimate 3000 UHPLC system (Dionex) coupled to a Q-Exactive Plus hybrid quadrupole-Orbitrap mass spectrometer. In order to identify the analytes, a heated electrospray interface was applied in both the positive and negative ionization modes.

The separation of the compounds was achieved using a Synergy Polar RP column (4 μm, 250 × 2.0 mm). The mobile phases consisted of water (A) and acetonitrile (B), both supplemented with 0.1% formic acid. A gradient elution profile was implemented, starting with an isocratic phase of 5% B for 1 min, followed by a gradient from 5 to 100% B over 25 min, an isocratic phase of 100% B for 5 min, a gradient from 100 to 5% B over 0.5 min, and an isocratic phase of 5% B for 11.5 min. The flow rate was 0.2 mL/min, and the injection volume was 3 µL. The column temperature was maintained at 25 °C.

The ion source parameters were set as follows: probe heater temperature 300 °C, ion transfer capillary temperature 320 °C, spray voltage 3.5 kV, sheath gas flow rate 30 arbitrary units, auxiliary gas flow rate 10 arbitrary units, and S-lens RF level 50 arbitrary units. Data acquisition employed a full-scan/data-dependent MS/MS method (Full MS/ddMS2) with a resolution of 70,000 for full scan MS spectra and 17,500 for ddMS2 scans. LC-HRMS data were acquired using Trace Finder 4.0 software and processed using Compound Discoverer™ 3.3 software. Further details about the Compound Discoverer settings are provided in the Supplementary Information.

## Data Availability

Data are contained within the article and Supplementary Materials.

## Supplementary Materials

The following supporting information can be downloaded at: https://www.mdpi.com/article/10.3390/toxins16090397/s1, Table S1: Overview of study material. Table S2: Dilution scheme for standard normalization. Table S3: Overview of RSD and target values from HPLC-DAD and LC-MS/MS measurements. Table S4: Overview of uncertainty contributions as well as acceptance range per standard. Table S5: Overview on molecular mass, annotation error, retention time, and fragmentation. Table S6: Compound Discoverer 3.3 workflow settings for ESI data.
