# Supplementary material for: Global Perspectives on Mycotoxin Reference Materials (Part I): Insights from Multi-Supplier Comparison Study Including Aflatoxin B1, Deoxynivalenol and Zearalenone
Source: Toxins (Basel). 2024 Sep 17;16(9):397. doi: 10.3390/toxins16090397 (PMC11435901; doi:10.3390/toxins16090397)
Supplement: Supplementary file 1 [file toxins-16-00397-s001.zip › toxins-3155882-supplementary.pdf]

Supplementary Materials

Global Perspectives on Mycotoxin Reference Materials (Part I): Insights from Multi-Supplier Comparison Study Including Aflatoxin B1, Deoxynivalenol and Zearalenone

David Steiner, Tibor Bartók, Michael Sulyok, András Szekeres, Mónika Varga, Levente Horváth and Helmut Rost

Table of Content

Table S 1: Overview of study material 2

Table S 2: Dilution scheme for standard normalization 3

Table S 3: Overview of RSD and target values from HPLC-DAD and LC-MS/MS measurements 4

Table S 4: Overview of uncertainty contributions as well as acceptance range per standard 5

Table S 5: Overview on molecular mass, annotation error, retention time, and fragmentation 6

Table S 6: Compound Discoverer 3.3 worflow settings for ESI data 8

Table S 1: Overview of study material

	Supplier	Product Code	Lot Number	Stock µg/mL	Solvent
Aflatoxin B1	Fermentek	SSAF	SSAF007	2.0	Acetonitrile
	Libios	FIA000194	AFB117101801	2.0	Acetonitrile
	Romer Labs	10006702	1000046205	2.0	Acetonitrile
	Merck	34029-2ML-R	BCCL0703	2.1	Acetonitrile
	JRC European Union	ERM - AC057	0636	3.8	Acetonitrile
	LVA	VE00008349	AFB202403001	9.6	Acetonitrile
	CPI International	Z-G34-013938-25	220307	10.0	Acetonitrile
	Trilogy	CTSL-131-5	211216-23342	10.2	Acetonitrile
	Oskar Tropitzsch	5500332	2C00G26	25.0	Acetonitrile
	Pribolab	STD#1042	2C10B20	100.0	Acetonitrile
Deoxynivalenol	JRC European Union	IRMM-315	0312	25.1	Acetonitrile
	Trilogy	CTSL-383-5	230502-24019	26.5	Acetonitrile
	Fermentek	SSDON	SSDON006	98.9	Acetonitrile
	Romer Labs	10006716	100004472	100.0	Acetonitrile
	Libios	FIA000239	DON17092701	100.0	Acetonitrile
	Oskar Tropitzsch	5500328	2C30C01	100.0	Acetonitrile
	Pribolab	STD#3102	2C30C01	100.0	Acetonitrile
	LVA	VE00008360	DON202403001	102.3	Acetonitrile
	Merck	34124-2ML	BCCL3588	103.5	Acetonitrile
	Merck	CRM46911	LRAD6006	200.0	Eth.Ac/MeOH 95:5
Zearalenone	JRC European Union	ERM-AC699	0587	10.0	Acetonitrile
	Trilogy	CTSL-422-5	221005-24015	10.2	Acetonitrile
	Merck	CRM46916	LRAD2894	50.0	Acetonitrile
	Fermentek	SSZ	SSZ006	99.5	Acetonitrile
	Romer Labs	10006718	1000020022	100.0	Acetonitrile
	Libios	FIA000311	ZEA17012401	100.4	Acetonitrile
	Oskar Tropitzsch	5500345	2C00E10	100.6	Acetonitrile
	Pribolab	STD#4012	2C00E10	100.6	Acetonitrile
	Merck	34126-2ML	BCCL0701	103.4	Acetonitrile
	LVA	VE00008369	ZON202403001	105.4	Acetonitrile

note: the order of the suppliers is randomized and does not correspond to the reference material producer order in the manuscript.

Table S 2: Dilution scheme for standard normalization

	Supplier	Conc. Stock µg/L	Stock Volume µL	Solvent Volume µL	final Conc. µg/L	Water %	Acetonitrile %
Aflatoxin B1	Fermentek	1997	250	2250	199.7	72.0%	28.0%
	Libios	2000	250	2250	200.0	72.0%	28.0%
	Romer Labs	2000	250	2250	200.0	72.0%	28.0%
	Merck	2090	239	2261	199.8	72.4%	27.6%
	JRC European Union	3790	264	4736	200.1	75.8%	24.2%
	LVA	9610	52	2448	199.9	78.3%	21.7%
	CPI International	10000	100	4900	200.0	78.4%	21.6%
	Trilogy	10230	98	4902	200.1	78.4%	21.6%
	Oskar Tropitzsch *	8347	120	4880	200.0	78.1%	21.9%
	Pribolab **	10000	100	4900	200.0	78.4%	21.6%
Deoxynivalenol	JRC European Union	25100	199	4801	1000.0	76.8%	23.2%
	Trilogy	26480	189	4811	999.9	77.0%	23.0%
	Fermentek	98945	50	4950	997.4	79.2%	20.8%
	Romer Labs	99980	50	4950	999.8	79.2%	20.8%
	Libios	100000	50	4950	1000.0	79.2%	20.8%
	Oskar Tropitzsch	100000	50	4950	1000.0	79.2%	20.8%
	Pribolab	100000	50	4950	1000.0	79.2%	20.8%
	LVA	102340	49	4951	998.8	79.2%	20.8%
	Merck	103500	48	4952	1001.9	79.2%	20.8%
	Merck ***	20000	250	4750	1000.0	76.4%	23.6%
Zearalenone	JRC European Union	9950	251	2249	999.0	72.0%	28.0%
	Trilogy	10150	246	2254	998.8	72.1%	27.9%
	Merck	50000	100	4900	1000.0	78.4%	21.6%
	Fermentek	99461	50	4950	998.6	79.2%	20.8%
	Romer Labs	100010	50	4950	1000.1	79.2%	20.8%
	Libios	100390	50	4950	999.9	79.2%	20.8%
	Oskar Tropitzsch	100600	50	4950	998.0	79.2%	20.8%
	Pribolab	100600	50	4950	998.0	79.2%	20.8%
	Merck	103400	48	4952	1000.9	79.2%	20.8%
	LVA	105370	47	4953	998.9	79.2%	20.8%

Dilution was carried out in Acetonitrile:Water (20:80)

* 1:3 dilution in Acetonitrile applied first

** 1:10 dilution in Acetonitrile applied first

*** 1:10 dilution in Acetonitrile applied first

Table S 3: Overview of RSD and target values from HPLC-DAD and LC-MS/MS measurements

	Supplier	LC-MS/MS					HPLC-DAD				
		Replicate 1 µg/L	Replicate 2 µg/L	Replicate 3 µg/L	RSD in %	Target Value in %	Replicate 1 µg/L	Replicate 2 µg/L	Replicate 3 µg/L	RSD in %	Target Value in %
Aflatoxin B1	RMP 1	197.4	196.9	198.2	0.33	98.8	193.1	193.3	192.8	0.13	96.5
	RMP 2	177.3	159.5	166.7	5.34	83.9	155.4	156.6	154.8	0.60	77.8
	RMP 3	205.6	203.6	199.6	1.50	101.5	203.0	204.2	204.3	0.35	101.9
	RMP 4	202.5	195.4	202.0	1.98	100.0	195.4	196.6	195.3	0.36	97.9
	RMP 5	222.1	214.4	212.2	2.41	108.1	220.2	219.5	221.0	0.34	110.1
	RMP 6	202.9	199.0	203.9	1.29	101.0	204.6	207.7	209.1	1.09	103.6
	RMP 7	207.7	201.4	204.3	1.54	102.2	201.1	209.8	210.8	2.58	103.6
	RMP 8	202.9	195.2	194.1	2.42	98.7	199.4	203.4	202.9	1.09	100.9
	RMP 9	199.8	200.0	207.1	2.05	101.1	205.2	202.6	206.7	1.01	102.4
	RMP 10	213.2	204.8	210.6	2.04	104.8	211.4	211.6	208.3	0.89	105.2
Deoxynivalenol	RMP 1	1078.8	1090.6	1082.6	0.44	108.8	1096.8	1070.3	1064.2	1.61	107.7
	RMP 2	836.0	812.8	816.1	0.40	81.6	827.5	793.4	791.3	2.53	80.4
	RMP 3	1167.5	1145.1	1130.5	0.69	113.6	1174.4	1151.1	1140.7	1.49	115.5
	RMP 4	1046.4	1019.5	1025.1	0.32	102.1	1029.3	996.4	1009.6	1.64	101.2
	RMP 5	987.8	985.6	972.7	0.71	97.8	1004.5	968.3	961.9	2.35	97.8
	RMP 6	1025.4	989.6	977.2	0.66	98.5	999.8	983.3	983.1	0.97	98.9
	RMP 7	966.9	961.1	959.7	0.51	96.3	1004.4	976.7	970.3	1.84	98.4
	RMP 8	1008.1	1008.0	1014.5	1.21	101.8	1031.9	1005.4	991.6	2.03	101.0
	RMP 9	1016.8	1035.1	1029.7	0.84	103.7	1049.2	1017.1	1019.1	1.75	102.8
	RMP 10	943.8	944.8	963.3	1.17	95.8	990.8	953.4	944.3	2.56	96.3
Zearalenone	RMP 1	983.8	933.8	956.9	2.61	95.8	938.6	946.0	945.3	0.44	94.3
	RMP 2	1004.0	960.1	974.7	1.86	98.0	953.4	961.0	964.4	0.59	96.0
	RMP 3	1126.0	1075.4	1075.7	2.17	109.2	1166.7	1169.8	1177.4	0.47	117.1
	RMP 4	993.1	943.7	945.5	2.38	96.1	965.4	955.8	965.0	0.56	96.2
	RMP 5	989.6	944.0	953.8	2.04	96.2	932.1	942.1	940.3	0.57	93.8
	RMP 6	1007.9	975.9	978.4	1.47	98.7	962.9	973.7	971.2	0.59	96.9
	RMP 7	1028.6	1002.9	1012.8	1.04	101.5	1007.6	1019.5	1014.1	0.59	101.4
	RMP 8	1044.9	990.0	1002.0	2.32	101.2	1002.5	1017.1	1013.5	0.75	101.1
	RMP 9	1006.3	986.1	964.7	1.72	98.6	968.4	981.6	982.7	0.81	97.8
	RMP 10	1082.3	1009.4	1047.6	2.85	104.6	1046.3	1056.0	1059.5	0.65	105.4

Table S 4: Overview of uncertainty contributions as well as acceptance range per standard

$$u = \sqrt{(u_{CoA/2})^2 + (u_{MS/MS})^2 + (u_{DAD})^2 + (u_{prep})^2}$$

	Supplier	u_{CoA} %	$u_{MS/MS}$ %	u_{DAD} %	u_{prep} %	$u_{combined}$ %	Lower Limit %	Upper Limit %
Aflatoxin B1	RMP 1	0.20	0.33	0.13	0.15	0.41	99.6	100.4
	RMP 2	2.90	5.34	0.60	0.06	5.75	94.3	105.7
	RMP 3	3.50	1.50	0.35	0.00	2.92	97.1	102.9
	RMP 4	3.00	1.98	0.36	0.06	2.93	97.1	102.9
	RMP 5	0.90	2.41	0.34	0.00	2.51	97.5	102.5
	RMP 6	2.01	1.29	1.09	0.10	2.21	97.8	102.2
	RMP 7	1.40	1.54	2.58	0.01	3.16	96.8	103.2
	RMP 8	1.20	2.42	1.09	0.00	2.79	97.2	102.8
	RMP 9	3.50	2.05	1.01	0.00	3.37	96.6	103.4
	RMP 10	6.55	2.04	0.89	0.05	5.14	94.9	105.1
Deoxynivalenol	RMP 1	0.04	0.44	1.61	0.26	1.69	98.3	101.7
	RMP 2	4.78	0.40	2.53	0.00	4.24	95.8	104.2
	RMP 3	0.79	0.69	1.49	0.00	1.74	98.3	101.7
	RMP 4	3.00	0.32	1.64	0.12	2.70	97.3	102.7
	RMP 5	1.50	0.71	2.35	0.00	2.67	97.3	102.7
	RMP 6	1.16	0.66	0.97	0.19	1.44	98.6	101.4
	RMP 7	1.40	0.51	1.84	0.00	2.15	97.8	102.2
	RMP 8	1.40	1.21	2.03	0.00	2.56	97.4	102.6
	RMP 9	2.93	0.84	1.75	0.02	2.84	97.2	102.8
	RMP 10	6.95	1.17	2.56	0.01	5.66	94.3	105.7
Zearalenone	RMP 1	0.04	2.61	0.44	0.14	2.65	97.4	102.6
	RMP 2	3.02	1.86	0.59	0.10	2.89	97.1	102.9
	RMP 3	0.75	2.17	0.47	0.01	2.29	97.7	102.3
	RMP 4	3.00	2.38	0.56	0.11	3.24	96.8	103.2
	RMP 5	0.40	2.04	0.57	0.00	2.13	97.9	102.1
	RMP 6	1.64	1.47	0.59	0.09	1.97	98.0	102.0
	RMP 7	1.39	1.04	0.59	0.20	1.56	98.4	101.6
	RMP 8	1.39	2.32	0.75	0.20	2.64	97.4	102.6
	RMP 9	2.59	1.72	0.81	0.01	2.64	97.4	102.6
	RMP 10	6.50	2.85	0.65	0.12	5.45	94.6	105.4

Table S 5: Overview on molecular mass, annotation error, retention time, and fragmentation

	Compound	Molecular formula	Molecular weight	Annotation error (ppm)	Retention Time (min)	Precursor ion	Fragment ions										AFB_1 (% of AFB1)	AFB_2 (% of AFB1)	AFB_3 (% of AFB1)	AFB_4 (% of AFB1)	AFB_5 (% of AFB1)	AFB_6 (% of AFB1)	AFB_7 (% of AFB1)	AFB_8 (% of AFB1)	AFB_9 (% of AFB1)	AFB_10 (% of AFB1)
Aflatoxin B1		C17 H14 O7	330,074	0,74	15,91	[M-H]-	329.0668 (100)	243.0298 (53)	258.0533 (35)	301.0718 (24)	285.0407 (19)	286.0484 (18)	290.0434 (18)	71.0136 (11)	257.0457 (9)	245.0455 (6)	0,13	0,02	0,39	0,03	0,76	0,12	0,12	0,90	0,90	2,67
		C16 H10 O7	314,043	0,81	15,94	[M-H]-	313.0355 (100)	269.0456 (58)	243.0299 (379	241.0508 (19)	285.0406 (16)	225.0559 (5)	215.0355 (3)	199.0396 (3)	197.061 (3)	213.0556 (3)	0,12	0,00	0,01	0,00	0,01	0,01	0,06	0,28	0,02	0,00
		C17 H12 O7	328,059	0,61	16,44	[M-H]-	283.0248 (100)	268.0377 (22)	299.0559 (19)	230.0221 (14)	244.0378 (13)	300.0276 (10)	312.0278 (10)	327.0512 (9)	239.0349 (9)	255.0230 (8)	0,00	0,00	0,00	0,01	0,00	0,01	0,13	0,45	0,05	0,02
		C18 H14 O7	342,075	3,53	16,64	[M-H]+	343.0821 (100)	313.0715 (50)	240.2318 (39)	325.0722 (38)	285.0750 (26)	297.0761 (15)	269.0809 (6)	310.0474 (69	253.0851 (6)	266.0571 (5)	0,01	0,01	0,02	0,09	0,01	1,33	0,14	0,01	0,02	0,01
		C17 H12 O7	328,060	4,21	17,10	[M-H]+	329.0672 (100)	311.0561 (59)	206.0573 (27)	283.0606 (23)	282.0524(11)	301.0719(11)	259.0596 (10)	245.0426 (6)	231.0649 (6)	178.0626 (6)	0,04	0,00	0,20	0,02	0,01	0,12	0,16	0,13	0,10	0,02
	Aflatoxin G1	C17 H12 O7	328,060	4,29	18,99	[M-Na]+	351.0490 (100)	322.0458 (6)	307.0238 (3)								0,01	0,01	0,01	0,44	0,11	0,58	0,01	0,02	0,01	0,09
	Aflatoxin B2	C17 H14 O6	314,081	4,97	19,15	[M-H]+	315.0873 (100)	287.0924 (12)	259.0586 (5)	68.4543 (5)	297.0775 (4)	98.0382 (1)	269.0796 (1)				0,09	0,19	0,05	1,16	0,71	0,84	0,01	0,23	0,01	0,71
	Aflatoxin B1	C17 H12 O6	312,064	3,18	20,01	[M-H]+	313.0716 (100)	285.0762 (34)	284.0684 (11)	298.0484 (7)	270.0523 (7)	257.0808 (5)	269.0443 (3)	243.0652 (3)	229.0860 (2)	242.0571 (2)	100,0	100,0	100,0	100,0	100,0	100,0	100,0	100,0	100,0	100,0
		C13 H24 O3 S3	324,089	0,45	21,35	[M-H]-	143.9016 (100)	79.9564 (25)	96.9601 (10)	112.9372 (2)							2,59	0,05	0,06	0,02	0,02	0,06	0,01	1,15	0,06	0,02
		C13 H24 O3 S4	356,061	0,62	22,95	[M-H]-	175.8736 (100)	95.9168 (45)	143.9016 (4)	79.9571 (3)	291.1101 (1)						0,43	0,07	0,08	0,03	0,02	0,07	0,01	1,61	0,08	0,03
																AFB2/AFG1	12,91	30,92	6,83	2,67	6,42	1,46	0,93	11,62	1,18	7,71
																>0.1	-1,00	-1,00	-1,00	-1,00	-1,00	-1,00	-1,00	-1,00	-1,00	-1,00
	**Compound**	**Molecular formula**	**Molecular weight**	**Annotation error (ppm)**	**Retention Time (min)**	**Precursor ion**	**Fragment ions**										**DON_1 (% of DON)**	**DON_2 (% of DON)**	**DON_3 (% of DON)**	**DON_4 (% of DON)**	**DON_5 (% of DON)**	**DON_6 (% of DON)**	**DON_7 (% of DON)**	**DON_8 (% of DON)**	**DON_9 (% of DON)**	**DON_10 (% of DON)**
Deoxynivalenol		C15 H21 N O5	295,143	3,45	4,69	[M-H]+	296.1503 (100)	136.0758 (30)	248.1281 (26)	260.1281 (20)	278.1392 (19)	138.055 (17)	202.1227 (15)	174.0913 (14)	230.1178 (14)	82.0657 (13)	16,99	0,77	19,04	36,13	0,03	0,00	9,92	6,80	0,03	0,54
		C15 H23 N O6	313,154	4,38	7,69	[M-H]+	314.1607 (100)	296.1503 (41)	249.1120 (22)	203.1066 (16)	231.1017 (12)	210.1126 (11)	181.0860 (10)	175.0752 (8)	189.0914 (8)		6,11	0,20	2,50	2,49	0,04	0,05	0,06	0,06	0,06	1,96
		C15 H20 O7	312,122	3,74	9,56	[M-H]+	313.1292 (100)	109.0656 (74)	125.0601 (68)	173.0963 (66)	145.1017 (56)	97.0658 (53)	121.0654 (50)	105.0708 (45)	161.0963 (43)	81.0704 (43)	0,15	0,07	0,20	0,09	6,65	0,24	0,26	0,23	0,02	0,12
	Deoxynivalenol	C15 H20 O6	296,127	3,22	10,71	[M-H]+	203.1066 (100)	175.0755 (80)	125.0600 (72)	231.1015 (54)	137.0597 (51)	69.0337 (50)	189.0911 (48)	161.06 (44)	173.0963 (38)	201.0909 (41)	100,0	100,0	100,0	100,0	100,0	100,0	100,0	100,0	100,0	100,0
		C14 H18 O5	266,116	0,28	11,06	[M-H]-	247.0976 (100)	229.0870 (94)	163.0766 (849	235.0977 (51)	265.1082 (49)	217.0871 (28)	203.0714 (17)	193.0870 (15)	214.0638 (12)	123.0452 (11)	1,71	0,63	0,99	2,16	0,99	3,06	0,94	0,90	0,51	2,09
		C15 H20 O5	280,132	1,52	11,08	[M-H]+	233.1171 (100)	125.0600 (78)	251.1277 (40)	97.0657 (29)	187.1117 (25)	109.0656 (23)	145.1017 (21)	173.0964 (20)	121.0652 (19)	263.1277 (16)	2,25	3,63	3,06	2,02	5,29	2,24	8,70	8,42	0,08	2,37
		C16 H24 O7	328,154	4,81	11,75	[M-H]+	181.0861 (100)	125.0601 (31)	249.1124 (28)	109.0656 (25)	93.0707 (24)	121.0653 (23)	105.0708 (19)	119.0860 (19)	135.0806 (18)	207.1021 (18)	16,01	0,49	13,57	12,92	6,28	0,08	0,02	0,02	0,02	25,04
		C14 H16 O5	264,100	0,13	11,93	[M-H]-	233.0819 (100)	263.0925 (51)	177.0557 (24)	215.0714 (15)	203.0714 (13)	205.0867 (8)	163.0764 (6)	187.0762 (5)	138.0324 (4)	245.0820 (4)	0,44	1,17	1,31	1,60	0,15	0,16	2,05	1,57	0,30	1,19
		C17 H24 O7	340,152	0,80	12,88	[M+FA-H]-	59.0134 (100)	163.0765 (65)	249.1137 (38)	231.1036 (34)	68.6250 (20)	177.0921 (13)	123.0453 (10)	99.0452 (9)	122.0372 (9)	339.1444 (9)	0,02	0,01	0,05	0,00	1,30	0,97	0,02	0,03	0,00	0,06
		C14 H14 O6	278,079		15,18	[M-H]-	219.0299 (100)	191.0350 (14)	277.0717 (12)	190.0269 (1)							0,00	0,01	0,00	0,00	2,68	0,71	0,03	0,03	0,00	0,01
	Zearalenone	C18 H22 O5	318,147	-0,03	21,01	[M-H]-	317.1395 (100)	175.0401 (25)	273.1498 (24)	149.0609 (9)	187.0403 (9)	131.0503 (8)	299.1291 (7)	161.0609 (6)	149.0245 (3)	289.1448 (3)	0,40	0,07	0,04	3,45	0,01	0,02	0,02	0,02	0,19	0,06

		C17 H15 Cl2 N3 O2	363,054	-0,03	21,72	[M-H]-	292.0178 (100)	242.0378 (76)	278.0144 (64)	294.1827 (44)	83.0249 (14)	260.0048 (12)	293.0256 (10)	55.0184 (9)	159.0565 (5)	166.0069 (5)	0,01	0,07	0,19	0,10	0,06	0,06	0,02	0,84	1,05	0,06
																>0.1	-1,00	-1,00	-1,00	-1,00	-1,00	-1,00	-1,00	-1,00	-1,00	-1,00

	Compound	Molecular formula	Molecular weight	Annotation error (ppm)	Retention Time (min)	Precursor ion	Fragment ions										ZON_1 (% of ZON)	ZON_2 (% of ZON)	ZON_3 (% of ZON)	ZON_4 (% of ZON)	ZON_5 (% of ZON)	ZON_6 (% of ZON)	ZON_7 (% of ZON)	ZON_8 (% of ZON)	ZON_9 (% of ZON)	ZON_10 (% of ZON)
Zearalenone		C18 H22 O6	334,142	0,38	17,76	[M-H]-	333.1345 (100)	161.0244 (27)	191.0350 (26)	133.0295 (19)	163.0401 (14)	289.1447 (13)	147.0452 (11)	203.0351 (7)	159.0451 (6)	315.1236 (5)	0,21	0,01	0,20	0,07	0,00	0,01	0,01	0,01	0,01	0,04
	Zearalanone	C18 H24 O5	320,162	-0,36	18,35	[M-H]-	319.1550 (100)	275.1653 (26)	301.1446 (7)	174.0322 (4)	160.0166 (3)	257.1548 (2)	291.1602 (2)	233.1547 (2)	175.0766 (1)		1,46	0,00	1,53	0,17	0,00	0,00	0,00	0,00	0,00	0,00
		C18 H22 O4	302,153	3,44	18,40	[M+H]+	175.0392 (100)	285.149 (95)	267.1379 (88)	177.0548 (84)	229.0860 (67)	215.0703 (61)	121.1017 (46)	201.0911 (45)	217.0859 (41)	135.1169 (40)	0,80	0,00	0,65	0,07	0,00	0,00	0,00	0,00	0,00	0,00
		C18 H20 O5	316,131	-0,04	19,79	[M-H]-	297.1130 (100)	175.0400 (79)	271.0975 (56)	315.1239 (44)	187.0400 (40)	201.0557 (34)	255.1026 (32)	149.0607 (28)	161.0608 (19)	271.1334 (19)	0,05	0,01	0,06	0,06	0,02	0,89	0,00	0,00	0,19	0,02
		C18 H24 O5	320,162	-0,34	20,36	[M-H]-	319.1551 (100)	275.1650 (4)	190.0271 (3)	146.0374 (1)							0,20	0,00	0,21	0,00	0,00	0,00	0,00	0,00	0,00	0,00
		C18 H21 N O8	379,127	-0,15	20,59	[M-H]-	189.0193 (100)	203.0351 (55)	331.1186 (40)	177.0193 (34)	332.1272 (25)	190.0272 (20)	310.1771 (15)	68.0602 (15)	304.1310 (14)	176.0116 (9)	0,00	0,29	0,00	0,00	0,00	0,00	0,00	0,00	0,00	0,00
	alpha-, or beta-zearalenol	C18 H24 O5	320,162	-0,92	20,91	[M-H]-	319.1551 (100)	275.1653 (29)	205.0873 (13)	301.1454 (8)	79.9571 (8)	70.6618 (2)	277.1072 (2)	161.0973 (2)			0,85	0,07	0,81	0,15	0,06	0,05	0,00	0,00	0,02	0,08
	Zearalenone	C18 H22 O5	318,147	-0,52	21,00	[M-H]-	317.1393 (100)	175.0400 (32)	273.1497 (25)	131.0502 (13)	149.0608 (10)	187.0401 (11)	299.1287 (9)	161.0608 (6)	149.0244 (4)	160.0167 (5)	100,0	100,0	100,0	100,0	100,0	100,0	100,0	100,0	100,0	100,0
		C17 H15 Cl2 N3 O2	363,054	0,07	21,70	[M-H]-	292.0177 (100)	278.0146 (67)	242.0379 (67)	294.1828 (19)	83.0249 (12)	293.0255 (11)	260.0047 (11)	55.0183 (7)			0,00	0,13	0,01	0,00	0,01	0,04	0,10	0,00	0,01	0,01
																ZAN/ZOL	0,58	91,71	0,53	0,84	101,53	114,68	17,48	20,13	97,31	64,97
																>0.1	-1,00	-1,00	-1,00	-1,00	-1,00	-1,00	-1,00	-1,00	-1,00	-1,00

Table S 6: Compound Discoverer 3.3 worflow settings for ESI data

**Select Spectra**	
**Peak Filters**	
S/N Threshold (FT-only)	1.5
**Detect Compounds**	
**General Settings**	
Mass Tolerance [ppm]	5 ppm
Min. Peak Intensity	10000
Min. # Scans per Peak	5
**Trace Detection**	
Max. Number of Gaps to Correct	2
Min. Number of Adjacent Non-Zeros	2
**Peak Detection**	
Chromatographic S/N Threshold	1.5
Gap Ratio Threshold	0.35
Max. Peak Width [min]	1
Min. Relative Valley Depth	0.1
**Isotope Pattern Detection**	
Group Isotopes	Br, Cl
Zig-Zag Index Threshold	0.2
Jaggedness Threshold	0.4
Modality Threshold	0.9
**Compound Detection**	
Ions	[2M+ACN+H]+1
	[2M+ACN+Na]+1
	[2M+H]+1
	[2M+K]+1
	[2M+Na]+1
	[M+ACN+2H]+2
	[M+ACN+H]+1
	[M+ACN+Na]+1
	[M+H]+1
	[M+H+K]+2
	[M+H+Na]+2
	[M+H-H2O]+1
	[M+K]+1
	[M+Na]+1
	[2M+FA-H]-1
	[2M-H]-1
	[M+FA-H]-1
	[M-H]-1
	[M-H-2H2O]-1
	[M-H-H2O]-1
Base Ions	[M+H]+1; [M-H]-1
**Group Compounds**	
**General Settings**	
Mass Tolerance	5 ppm
RT Tolerance [min]	0.2
Peak Rating Filter	
Peak Rating Threshold	4
Number of Files	1
**Fill Gaps**	
**General Settings**	
Mass Tolerance	5 ppm
S/N Threshold	1.5

**Apply SERRF QC Correction**	
**General Settings**	
Min. QC Coverage [%]	50
Max. QC Area RSD [%]	30
Max. Corrected QC Area RSD [%]	25
Max. # Files Between QC Files	15
**Mark Background Compounds**	
**General Settings**	
Max. Sample/Blank	5
**Assign Compound Annotations**	
**General Settings**	
Mass Tolerance	5 ppm
**Data Sources**	
Data Source #1	mzCloud Search
Data Source #2	Predicted Compositions
Data Source #3	MassList Search
Data Source #4	ChemSpider Search
Data Source #5	Metabolika Search
**Search mzCloud**	
**General Settings**	
Precursor Mass Tolerance 10 ppm	10 ppm
FT Fragment Mass Tolerance 10 ppm	10 ppm
IT Fragment Mass Tolerance 0.4 Da	0.4 Da
Library Autoprocessed	Reference
**Search ChemSpider**	
**Search Settings**	
Database(s) BioCyc	Human Metabolome Database; KEGG
Mass Tolerance	5 ppm
**Predict Compositions**	
**Prediction Settings**	
Mass Tolerance	5 ppm
Min. Element Counts	C H
Min. RDBE	0
Max. RDBE	40
Min. H/C	0.1
Max. H/C	3.5
Max. # Candidates	10
Max. # Internal Candidates	200
